# Relationship between non-alcoholic fatty liver and progressive fibrosis and serum 25-hydroxy vitamin D in patients with type 2 diabetes mellitus

**DOI:** 10.1186/s12902-024-01640-2

**Published:** 2024-07-10

**Authors:** Jing-Xian Fang, Yu Han, Jian Meng, Hui-Ming Zou, Xue Hu, Yue-Xia Han, Fang Huang, Qing Gu, Sui-Jun Wang

**Affiliations:** Endocrinology and Metabolism, Yangpu District Shidong Hospital of Shanghai, No.999, Shiguang Road, Yangpu District, Shanghai, 200438 China

**Keywords:** Type 2 diabetes mellitus, Non-alcoholic fatty liver disease, Progressive liver fibrosis, 25-hydroxyvitamin D

## Abstract

**Objective:**

We aimed to analyze the relationship between non-alcoholic fatty liver and progressive fibrosis and serum 25-hydroxy vitamin D (25(OH)D) in patients with type 2 diabetes mellitus.

**Methods:**

A total of 184 patients with T2DM who were hospitalized in the Department of Endocrinology of the ShiDong Clinical Hospital between January 2023 and June 2023 were selected. We compared review of anthropometric, biochemical, and inflammatory parameters and non-invasive scores between groups defined by ultrasound NAFLD severity grades.We determine the correlation between 25(OH)D and FLI and FIB-4 scores, respectively.

**Results:**

Statistically significant differences were seen between BMI, WC, C-peptide levels, FPG, ALT, serum 25(OH)D, TC, HDL, lumbar spine bone density, FLI, and FIB-4 in different degrees of NAFLD. Multivariate logistic regression analysis showed that 25(OH)D (OR = 1.26, *p* = 0.001), age (OR = 0.93, *P* < 0.001) and BMI (OR = 1.04, *p* = 0.007) were independent predictors of NAFLD in patients with T2DM.

**Conclusions:**

This study revealed the correlation between serum 25(OH)D levels and NAFLD in patients with T2DM. We also demonstrated that serum 25(OH)D levels were negatively correlated with FLI/FIB-4 levels in patients with T2DM with NAFLD, suggesting that vitamin D deficiency may promote hepatic fibrosis progression in T2DM with NAFLD.

## Introduction

Nonalcoholic Fatty Liver Disease (NAFLD) is a liver disease characterized by an excessive accumulation of fat in the liver but not associated with excessive alcohol consumption [1]. Non-alcoholic steatohepatitis (NASH) is a severe type of NAFLD characterized pathologically by hepatic steatosis, balloon-like degeneration of hepatocytes, and lobular inflammation [2]. They are related to abnormal liver enzyme levels, insulin resistance, type 2 diabetes mellitus (T2DM), and increased cardiovascular risk [3]. The global prevalence of NAFLD and NASH is estimated to be around 25% and 5%, respectively, and is increasing [4]. NASH is indeed a serious health problem, and its progression can lead to a variety of serious complications. As NASH continues to develop, the damage to the liver becomes progressively worse, eventually leading to the development of cirrhosis. Liver failure is also one of the serious complications that may occur in the later stages of NASH development [5].

Steatosis and fibrosis are common pathologic processes in liver diseases, and their early detection and monitoring of their course are crucial for patient treatment and management. Traditional diagnostic methods for liver diseases, such as liver biopsy, although highly accurate, are invasive and carry certain risks. Therefore, in recent years, noninvasive scoring methods have received widespread attention and application. Non-invasive scoring methods are mainly based on biochemical parameters, epidemiological data, and anthropometric indicators, combined with non-invasive imaging techniques such as ultrasound and magnetic resonance to assess the degree of hepatic steatosis and fibrosis. These methods can help in the early detection of liver lesions, monitoring of disease progression, and assessment of treatment efficacy [6–8]. The European Association for the Study of Diabetes (EASD) has approved the use of the Fatty Liver Index (FLI) and the Fibrosis-4 Index (FIB-4) to assess liver fibrosis [9].

Approximately 60% of T2DM patients suffer from NAFLD [10]. Several studies have emphasized the strong interaction between NAFLD and T2DM and described a complex bidirectional relationship [11].

Studies have found that people with fatty liver often have abnormal levels of vitamins. Vitamins play a role in the development of NAFLD [12], but the roles and mechanisms of many vitamins in NAFLD are not clear at present.

Vitamin D is a steroid hormone that plays an important role in the regulation of bone and calcium homeostasis [13]. In addition to its classical role in bone health, vitamin D has recently been shown to have a variety of biological roles in different cell and tissue types, regulating cell proliferation, differentiation and immune regulation [14]. Indeed, many studies have shown that low circulating levels of vitamin D are associated with the development of liver fibrosis in patients with various chronic liver diseases (CLDs) [15, 16]. Vitamin D also play an important role in modifying the risk of T2DM, mainly in terms of its mediation of beta-cell function, insulin sensitivity, and systemic inflammatory responses [17].

Currently, there are few studies on the relationship between 25(OH)D levels and the degree of liver fibrosis in patients with T2DM combined with NAFLD. We hypothesized that serum 25(OH)D levels may play a role in the pathogenesis of NAFLD and its progression to fibrosis in T2DM patients.

## Materials and methods

### Patients and study design

184 patients with T2DM who were hospitalized in the Department of Endocrinology of the ShiDong Clinical Hospital between January 2023 and June 2023 were selected, and the diagnosis of T2DM was based on the 1999 WHO Diagnostic and Classification Criteria.

Inclusion Criteria: 1)Age ≥ 18 years. 2) T2DM conformed to 1999 WHO Diagnostic Criteria. 3) Ultrasound Diagnosis Grading Criteria: assessed according to the degree of echo weakening in the liver by color ultrasound examination. Liver ultrasound was performed by the same ultrasonographer experienced in liver disease ultrasonography aiming to obtain uniform NAFLD stratification and to avoid interobserver variations in findings. On each study participant, liver ultrasound was performed by a Toshiba Xario SSA-660 A (Toshiba, Japan) device, equipped with a 5 MHz convex probe. The NAFLD grade description is based on the ultrasonographic finding. There are three grades of NAFLD based on visual analysis. Grade 1 NAFLD presents with increased liver echogenicity, grade 2 NAFLD with the echogenic liver obscuring the echogenic walls of the portal venous branches, and grade 3 NAFLD diaphragmatic out line is obscured.The levels of 25(OH)D in the patients were divided into three grades: <20 ng/mL, 20–30 ng/mL and > 30 ng/mL.

Exclusion Criteria: For patients who drink a lot of alcohol, the amount of alcohol consumed should equate to the amount of alcohol consumed, which is more than 140 g/week for men and more than 70 g/week for women; use of medications that affect vitamin D levels; T1DM, special type diabetes mellitus, acute complications of diabetes mellitus; pregnant and lactating women; ethanol-induced or drug-induced liver disease, autoimmune disease, viral hepatitis, cholestasis, and metabolic liver disease, hepatomegaly; total parenteral nutrition; autoimmune liver disease; thyroid disease; rheumatoid immune disease.

### Data collection

General clinical information [gender, age, waist circumference (WC), height, weight, blood pressure was collected from the medical history. Blood measurements included fasting plasma glucose(FPG), fasting C-peptide, fasting insulin, glycosylated hemoglobin (HbA1c), 25-hydroxy vitamin D[25(OH)D], total cholesterol (TC), triglycerides (TG), high-density lipoprotein (HDL), low-density lipoprotein cholesterol (LDL-C), alanine aminotransferase (ALT), aspartate aminotransferase (AST), uric acid (UA), creatinine (Cr), urea nitrogen (BUN), and other biochemicals. Body mass index (BMI) is determined by the formula weight (kg)/height (m)2.

All patients were grouped according to their diagnosis after a color ultrasound of the liver. Lumbar spine bone mineral density was measured using dual-energy X-bone absorptiometry in the enrolled patients.

### Statistical analysis

The statistical analysis of this study was conducted using IBM SPSS statistics software Version 26. Measurement data were subjected to K-S normality test, and the data conforming to normal distribution were expressed as mean ± standard deviation (x ± s).Comparisons between groups were analyzed by one-way ANOVA.We used the parametric Pearson test and the nonparametric Spearman test to determine correlation. Multivariate logistic regression modeling were performed with the effect of several predictors on ultrasound grading results in nonalcoholic fatty liver disease.

## Results

A total of 184 T2DM patients with NAFLD were enrolled in the study. 89 (48%) of the 184 patients with NAFLD had mild fatty liver, 55 (30%) had moderate fatty liver, and 40 (22%) had severe fatty liver. Table 1 shows the differences in anthropometric, metabolic profile and non-invasive scores of steatosis between the groups. Statistically significant differences were seen between BMI, WC, C-peptide levels, FPG, ALT, serum 25(OH)D, TC, HDL, lumbar spine bone density, FLI, and FIB-4 in different degrees of NAFLD.

**Table 1:** A comparative review of biochemical and inflammatory parameters and non-invasive scores between groups defined by ultrasound NAFLD severity grades

Characteristics	Grade 1 of liver steatosis (*n* = 89)	Grade 2 of liver steatosis (*n* = 55)	Grade 3 of liver steatosis (*n* = 40)	F	*P*
BMI (kg/m2 )	25.61 ± 2.13	26.50 ± 1.83	27.67 ± 1.62ab	6.049	0.003
WC (cm)	90.43 ± 7.75	102.12 ± 7.66	102.21 ± 8.01ab	94.200	0.000
HbA1c (%)	7.55 ± 0.80	7.47 ± 0.53	7.47 ± 0.53	0.225	0.799
C-peptide (ng/ml)	2.43 ± 0.58	4.13 ± 1.20	4.99 ± 0.61ab	48.9	0.000
FPG (mmol/l)	7.67 ± 1.23	6.41 ± 1.00	7.40 ± 0.63ab	4.49	0.009
Platelet count(*10^9^ /L)	132 ± 43	144 ± 29	138 ± 38	7.431	0.099
ALT (U/L)	24.70 ± 10.36	32.33 ± 11.82	54.55 ± 4.42ab	18.05	0.000
AST (U/L)	20.91 ± 3.61	19.05 ± 2.90	31.20 ± 3.64	0.834	0.438
γGT (U/L)	22 ± 13	69 ± 20	122 ± 31	5.833	0.001
25(OH)D (ng/ml)	35.69 ± 6.10	26.70 ± 3.10a	14.53 ± 6.26ab	7.17	0.001
PTH (pg/ml)	37.67 ± 11.23	36.41 ± 14.00	37.40 ± 10.63	4.99	0.339
TC (mmol/l)	6.63 ± 1.39	7.67 ± 1.23	7.67 ± 1.23ab	4.208	0.018
TG (mmol/l)	2.61 ± 0.71	2.48 ± 0.69	2.54 ± 0.62	0.411	0.664
HDL (mmol/l)	1.38 ± 0.28	1.72 ± 0.39	1.66 ± 0.38ab	9.800	0.000
LDL (mmol/l)	3.84 ± 0.80	3.59 ± 0.69	3.73 ± 0.99	1.077	0.345
FLI	57.74 ± 17.30	75.89 ± 9.65	89.86 ± 9.56ab	5.286	0.007
FIB−4	1.67 ± 0.44	1.95 ± 0.99	2.46 ± 0.23ab	4.206	0.018
lumbar spine bone density (g/cm^2^)	0.99 ± 0.44	0.87 ± 0.61	0.75 ± 0.59ab	3.734	0.006

*BMI* body mass index, *WC* waist circumference, *HbA1c* glycated hemoglobin, *25(OH)D* 25-hydroxy vitamin D, *FPG* fasting plasma glucose, *ALT* alanine transaminase, *AST* aspartate aminotransferase, *γGT* gamma-glutamyl transferase, *PTH* Parathyroid Hormone, *TC* total cholesterol, *TG* triglycerides, *HDL* high density lipoprotein, *LDL* low density lipoprotein, *FLI* Fatty liver index score, FIB-4- Fibrosis-4 score

a indicates *P*<0.05, compare with hepatic steatosis grade 1; b indicates *P*<0.05, compare with hepatic steatosis grade 2。

Multivariate logistic regression analysis was performed with NAFLD as the dependent variable and anthropometric, metabolic profile and non-invasive scores of steatosis as independent variables. The results showed that 25(OH)D (OR = 1.26, *p* = 0.001), age (OR = 0.93, *P* < 0.001) and BMI (OR = 1.04, *p* = 0.007) were independent predictors of NAFLD in patients with T2DM (Table 2).

**Table 2:** Multivariate logistic regression analysis of factors associated with NAFLD in T2DM patients

Covariate	Univariate analysis			Multivariate analysis		
	OR	95% CI	*P*	OR	95% CI	*P*
Age (years)	1.02	0.99–1.07	< 0.001	0.93	0.89–0.95	< 0.001
Sex						
Male						
Female	0.68	0.55–0.87	0.465			
BMI (kg/m2 )	1.12	1.02–1.14	0.004	1.04	1.00-1.07	0.007
WC (cm)	1.13	1.07–1.15	0.038	0.83	0.79–0.85	0.344
HbA1C(%)	0.93	0.88–0.97	0.067			
C-peptide (ng/ml)	0.73	0.69–0.78	0.694			
FPG(mmol/l)	0.67	0.61–0.73	0.072			
ALT(U/L)	0.66	0.62–0.70	0.029			
AST(U/L)	0.73	0.68–0.76	0.018			
25(OH)D(ng/ml)	1.17	1.14–1.23	0.003	1.26	1.22–1.29	0.001
PTH(pg/ml)	0.13	0.07–0.16	0.562			
TC(mmol/l)	0.46	0.42–0.49	0.849			
TG(mmol/l)	0.69	0.63–0.73	0.712			
HDL-C(mmol/l)	0.34	0.30–0.37	0.088			
LDL-C(mmol/l)	1.28	0.24–0.33	0.672			
FLI	1.18	1.14–1.22	0.012	0.74	0.71–0.77	0.027
FIB-4	1.23	1.19–1.27	0.025	1.09	1.06–1.12	0.011

*BMI* body mass index, *WC* waist circumference, *HbA1c* glycated hemoglobin, *25(OH)D* 25-hydroxy vitamin D, *FPG* fasting plasma glucose, *ALT* alanine transaminase, *AST* aspartate aminotransferase, *γGT* gamma-glutamyl transferase, *PTH* Parathyroid Hormone, *TC* total cholesterol *TG* triglycerides, *HDL* high density lipoprotein, *LDL* low density lipoprotein, *FLI* Fatty liver index score, *FIB-4* Fibrosis-4 score

a indicates *P*<0.05, compare with hepatic steatosis grade 1; b indicates *P*<0.05, compare with hepatic steatosis grade 2

**Fig. 1:**
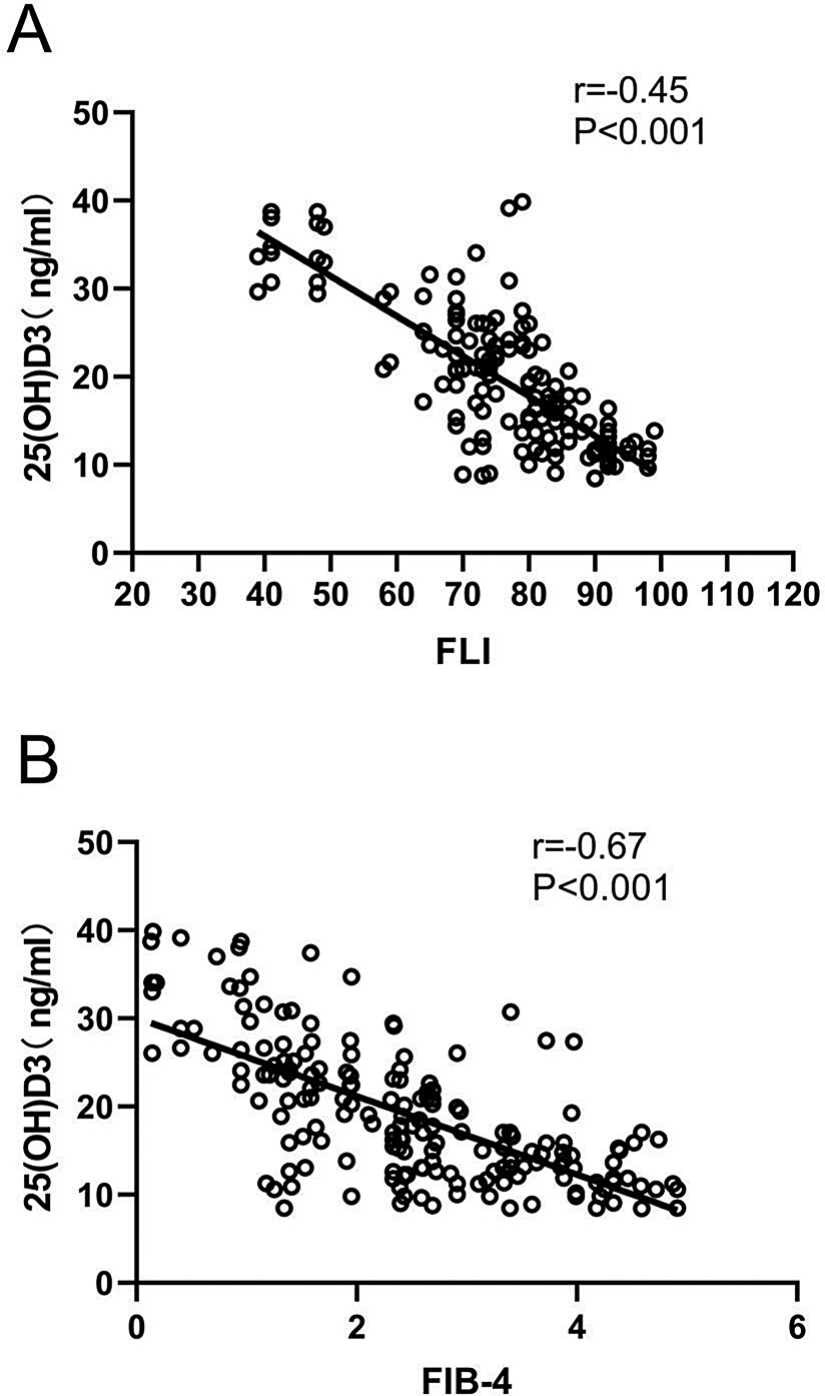
Showed the correlation between (**A**) 25(OH)D and Fibrosis- 4 (FIB-4) score and (**B**) 25(OH)D and fatty liver index (FLI) score

Correlation analysis revealed that there occurs a negative association between 25(OH)D and FLI (*r* = -0.45, *P* < 0.001) (Fig. 1A). As shown in Fig. 1B, there was negative association between 25(OH)D and FIB-4 (*r* = -0.67, *P* < 0.001).

## Discussion

The direct mechanism between 25(OH)D deficiency and liver fibrosis has not yet been clearly revealed by scientific research. However, we can explore the relationship between the two in terms of their respective effects and possible interactions. In the ANOVA, there was a significant difference in 25(OH)D levels between the different groups. In correlation analysis, we have found a significant negative correlation between 25(OH)D and FIB-4 and FLI. Serum 25(OH)D was an influential factor for NAFLD in multivariate logistic regression analysis. 25(OH)D activation exerts anti-inflammatory and anti-fibrotic effects in the liver by modulating the immune system, inhibiting the expression of pro-fibrotic inflammatory mediators such as platelet-derived growth factor (PDGF) and transforming growth factor β (TGF-β), and promoting the expression of collagen, α-smooth muscle actin, and tissue inhibitor of metalloproteinase-1 [18, 19]. However, once cirrhosis develops, vitamin D loses its antifibrotic protective effect [20].

Some studies suggest that 25(OH)D levels may decline as the severity of NAFLD increases [21]. This decline may be related to impaired liver function, as the liver is an important organ for 25(OH)D synthesis and metabolism [22].Liangpunsakul S et al. found that serum vitamin D levels were significantly lower in those with unexplained elevations in ALT than in controls. In this article, we observed significant differences in ALT between subgroups with different degrees of NAFLD, besides, in a multiple logistic regression analysis, ALT was also found to have a significant effect on the severity of NAFLD [23]. However, in a meta-analysis based on six cross-sectional biopsy studies, Saberi et al. did not find a significant difference between 25(OH)D levels and the degree of liver fibrosis in patients with nonalcoholic fatty liver disease (NAFLD) [24].

In this study, FLI and FIB-4 were statistically different between groups determined by ultrasound NAFLD severity. Therefore, we concluded that FLI and FIB-4 scores were significantly associated with ultrasound NAFLD stratification. We used both of these indicators to score the degree of hepatic fibrosis in T2DM patients. We found that 25(OH)D was negatively correlated with FIB4 and FLI, all of which suggest that 25(OH)D deficiency may promote the progression of hepatic fibrosis in T2DM patients with NAFLD [25].

Our study showed that the was lumbar spine bone density significantly associated with ultrasound NAFLD stratification. The results of this study suggest a correlation between NAFLD and osteoporosis. However, our findings do not support a causal role of osteoporosis on NAFLD. This finding suggests that there may be bone-liver interactions, which are worthy of further study [26].

There was a statistically significant difference in BMI between groups. BMI showed a significant association with NAFLD in both univariate and multivariate analyses. The higher the BMI, the greater the risk of NAFLD (OR 1.12 in univariate analysis and 1.04 in multivariate analysis). This suggests that obesity is a significant risk factor for NAFLD. The increased prevalence of NAFLD is associated with a rising trend in obesity, especially among morbidly obese patients [27–29].

Among the glycemic-related indices, the levels of C-peptide and fasting glucose (FPG) varied with increasing grades of NAFLD. C-peptide levels were significantly higher in grades 2 and 3, whereas FPG levels were relatively high in grades 1 and 3. These changes may be related to insulin resistance due to hepatic steatosis. Alfadda et al. investigated the prevalence of NAFLD in patients with T2DM using transient elastography and found that 80.8% of T2DM patients had steatosis, of which 82.3% had severe steatosis and 17.6% had mild to moderate steatosis [30]. 25(OH)D deficiency promotes the development and progression of NAFLD by causing insulin resistance, increasing hepatic resistin gene expression, and upregulating hepatic steatosis and oxidative stress gene expression [31].

Overall, further studies are needed to clarify the apparent differences in 25(OH)D levels between different degrees of NAFLD at this time. Future studies could conduct larger surveys and clinical trials in patients with different degrees of NAFLD to more accurately assess the relationship between 25(OH)D levels and NALD severity. There is also a need to further explore the mechanism of 25(OH)D’s role in the pathogenesis and treatment of NAFLD and how to improve the condition of patients with NAFLD by adjusting 25(OH)D levels. This will help provide new ideas and methods for the prevention and treatment of NAFLD.

## Conclusion

This study revealed the correlation between serum 25(OH)D levels and NAFLD in patients with T2DM. We also demonstrated that serum 25(OH)D levels were negatively correlated with FLI/FIB-4 levels in patients with T2DM with NAFLD, suggesting that vitamin D deficiency may promote hepatic fibrosis progression in T2DM with NAFLD.

## Data Availability

All data supporting the findings of this study are available within the paper and its Supplementary Information.

## References

[1] Petroni ML et al. Moderate Alcohol Intake in non-alcoholic fatty liver disease: to drink or not to drink? Nutrients, 2019. 11(12). 10.3390/nu11123048 PMC6950084 31847199

[2] Huby T, Gautier EL (2022). Immune cell-mediated features of non-alcoholic steatohepatitis. Nat Rev Immunol.

[3] Hassen G (2022). Nonalcoholic fatty liver disease: an emerging modern-day risk factor for Cardiovascular Disease. Cureus.

[4] Younossi ZM (2019). The global epidemiology of NAFLD and NASH in patients with type 2 diabetes: a systematic review and meta-analysis. J Hepatol.

[5] Schuster S (2018). Triggering and resolution of inflammation in NASH. Nat Rev Gastroenterol Hepatol.

[6] Wieckowska A, Feldstein AE (2008). Diagnosis of nonalcoholic fatty liver disease: invasive versus noninvasive. Semin Liver Dis.

[7] Loomba R, Adams LA (2020). Advances in non-invasive assessment of hepatic fibrosis. Gut.

[8] Bedogni G (2006). The fatty liver index: a simple and accurate predictor of hepatic steatosis in the general population. BMC Gastroenterol.

[9] Di Mauro S et al. Clinical and molecular biomarkers for diagnosis and staging of NAFLD. Int J Mol Sci, 2021. 22(21). 10.3390/ijms222111905 PMC8585051 34769333

[10] Arteh J, Narra S, Nair S (2010). Prevalence of vitamin D deficiency in chronic liver disease. Dig Dis Sci.

[11] Tanase DM et al. The Intricate Relationship between Type 2 Diabetes Mellitus (T2DM), Insulin Resistance (IR), and Nonalcoholic Fatty Liver Disease (NAFLD) J Diabetes Res, 2020. 2020: p. 3920196. 10.1155/2020/3920196 PMC7424491 32832560

[12] Abe RAM (2021). The role of vitamins in non-alcoholic fatty liver disease: a systematic review. Cureus.

[13] Anderson PH, Turner AG, Morris HA (2012). Vitamin D actions to regulate calcium and skeletal homeostasis. Clin Biochem.

[14] Hossein-nezhad A, Holick MF (2013). Vitamin D for health: a global perspective. Mayo Clin Proc.

[15] Petta S (2010). Low vitamin D serum level is related to severe fibrosis and low responsiveness to interferon-based therapy in genotype 1 chronic hepatitis C. Hepatology.

[16] Terrier B (2011). Low 25-OH vitamin D serum levels correlate with severe fibrosis in HIV-HCV co-infected patients with chronic hepatitis. J Hepatol.

[17] Mitri J, Pittas AG (2014). Vitamin D and diabetes. Endocrinol Metab Clin North Am.

[18] Timms PM (2002). Circulating MMP9, vitamin D and variation in the TIMP-1 response with VDR genotype: mechanisms for inflammatory damage in chronic disorders?. QJM.

[19] Targher G (2007). Associations between serum 25-hydroxyvitamin D3 concentrations and liver histology in patients with non-alcoholic fatty liver disease. Nutr Metab Cardiovasc Dis.

[20] Yuan S, Larsson SC (2023). Inverse Association between serum 25-Hydroxyvitamin D and nonalcoholic fatty liver disease. Clin Gastroenterol Hepatol.

[21] Ciardullo S (2023). Low 25 (OH) vitamin D levels are associated with increased prevalence of nonalcoholic fatty liver disease and significant liver fibrosis. Diabetes Metab Res Rev.

[22] Cai J (2020). Correlation between serum 25-OH vitamin D expression and non-alcoholic fatty liver disease. Exp Ther Med.

[23] Liangpunsakul S, Chalasani N (2011). Serum vitamin D concentrations and unexplained elevation in ALT among US adults. Dig Dis Sci.

[24] Saberi B (2018). Vitamin D levels do not predict the stage of hepatic fibrosis in patients with non-alcoholic fatty liver disease: a PRISMA compliant systematic review and meta-analysis of pooled data. World J Hepatol.

[25] Taban L et al. Vitamin D status and Steatohepatitis in obese Diabetic and non-diabetic patients. J Clin Med, 2022. 11(18). 10.3390/jcm11185482 PMC9503920 36143129

[26] Cui A (2023). Causal association of NAFLD with osteoporosis, fracture and falling risk: a bidirectional mendelian randomization study. Front Endocrinol (Lausanne).

[27] Quek J (2023). Global prevalence of non-alcoholic fatty liver disease and non-alcoholic steatohepatitis in the overweight and obese population: a systematic review and meta-analysis. Lancet Gastroenterol Hepatol.

[28] Barranco-Fragoso B (2022). Identification of hepatic dendritic cells in liver biopsies showing steatosis in patients with metabolic dysfunction-Associated fatty liver Disease (MAFLD) Associated with obesity. Med Sci Monit.

[29] Chen X (2022). Associations between abdominal obesity indices and nonalcoholic fatty liver disease: Chinese visceral Adiposity Index. Front Endocrinol (Lausanne).

[30] Alfadda AA (2022). Transient elastography for the prevalence of non-alcoholic fatty liver disease in patients with type 2 diabetes: evidence from the CORDIAL cohort study. Saudi J Gastroenterol.

[31] Contreras-Bolívar V et al. Mechanisms involved in the relationship between Vitamin D and insulin resistance: impact on clinical practice. Nutrients, 2021. 13(10). 10.3390/nu13103491 PMC8539968 34684492

